# Characterization of WO_3_/Silicone Rubber Composites for Hydrogen-Sensitive Gasochromic Application

**DOI:** 10.3390/molecules29153499

**Published:** 2024-07-26

**Authors:** Lin Wang, Ke Yang, Ping Yu, Huan Liu, Qingli Cheng, Anfeng Yu, Xinmei Liu, Zhe Yang

**Affiliations:** 1 College of Chemistry and Chemical Engineering, China University of Petroleum (East China), Qingdao 266580, China; wangl.qday@sinopec.com; 2 State Key Laboratory of Chemical Safety, Sinopec Research Institute of Safety and Engineering Co., Ltd., Qingdao 266000, China; kikiwow@163.com (K.Y.); yup.qday@sinopec.com (P.Y.); liuh.qday@sinopec.com (H.L.); chengql.qday@sinopec.com (Q.C.); yuaf.qday@sinopec.com (A.Y.)

**Keywords:** hydrogen-sensitive, gasochromic, silicone rubber, tungsten trioxide

## Abstract

WO_3_ and silicone rubber (SR)-based gasochromic composites were fabricated to detect hydrogen leaks at room temperature. WO_3_ rod-like nanostructures were uniformly distributed in the SR matrix, with a particle size of 60–100 nm. The hydrogen permeability of these composites reached 1.77 cm^3^·cm/cm^2^·s·cm_Hg_. At a 10% hydrogen concentration, the visible light reflectance of the composite decreased 49% during about 40 s, with a color change rate of 6.4% s^−1^. Moreover, the composite detected hydrogen concentrations as low as 0.1%. And a color scale was obtained for easily assessing hydrogen concentrations in the environment based on the color of composites. Finally, the composite materials as disposable sensors underwent testing at several Sinopec hydrogen refueling stations.

## 1. Introduction

Hydrogen energy is obtaining significant attention as a clean energy solution to address global warming concerns [1,2]. However, the highly explosive nature of hydrogen, characterized by its wide combustion range and low ignition energy, poses a critical challenge to the safe operation of hydrogen energy systems [3,4]. The installation of hydrogen energy equipment, along with the integration of highly effective leakage detectors, holds paramount significance in ensuring the safety and reliability of our energy infrastructure. This approach is crucial for promoting sustainable energy development and safeguarding the environment. Essential features for detectors include cost-effectiveness, explosion resistance, and accurate detection of hydrogen leaks across wide areas. This capability is essential for establishing a secure and dependable hydrogen energy network [5,6]. Hydrogen-sensitive gasochromic materials offer a significant solution, as they undergo noticeable color changes in the presence of hydrogen, enabling visual identification of hydrogen leakage locations. This feature allows for timely detection and mitigation of potential safety hazards [7,8]. Compared to traditional leakage detectors, hydrogen-sensitive gasochromic materials offer several advantages. Firstly, they work without external voltage, relying solely on hydrogen-induced color changes, thereby eliminating risks associated with external power sources. Secondly, these materials provide precise finding of hydrogen leaks, enhancing detector accuracy to prevent safety incidents. With hydrogen energy expansion, hydrogen-sensitive gasochromic materials are receiving increased research attention.

As a typical hydrogen-sensitive gasochromic material, WO_3_ possesses unique advantages in the hydrogen-detecting field [9,10,11,12]. Compared to other hydrogen-sensitive materials, WO_3_ exhibits at least three distinct advantages. Firstly, when exposed to hydrogen, WO_3_ undergoes an obvious coloring process, transitioning gradually from light yellow to deep blue. This noticeable color change enhances its optical sensitivity. Secondly, WO_3_ has excellent chemical stability, maintaining its complete structure even during subsequent processing. This characteristic ensures a longer lifespan and more reliable performance in practical applications. Thirdly, WO_3_ is cost-effective and can be synthesized through simple chemical reactions, making it advantageous for large-scale production. This characteristic can reduce production costs for widespread utilization in the hydrogen-detecting field.

The utilization of WO_3_ as a metal oxide has challenges in shaping the material. In contrast, polymer-based composite detectors have many advantages. Polymer-based materials, such as polyaniline [13,14], polypyrrole [15,16], polythiophene [17], polyvinyl pyrrolidone [18,19,20], polyvinyl alcohol [21,22,23] and polyvinylidene fluoride [24], have the characteristics of low cost and easy processing, making them extensively utilized in the detecting field. These composites combine the advantages of polymer and metal oxide, which can effectively improve the performance of the detectors and expand their application in various fields. Using the RF magnetron sputtering system, the WO_3_ @ PET film, which demonstrates outstanding gasochromic performance, was obtained [8]. After 10 min under a hydrogen atmosphere, the film exhibited a 50% change in light transmittance. Similarly, the flexible hydrogen detector also reported that WO_3_ nanoparticles were deposited onto the flexible polyimide tape by electrostatic spray deposition [25]. This flexible hydrogen detector showed an obvious color change within 10 min under the hydrogen atmosphere. Despite these composites exhibiting remarkable gasochromism to hydrogen, they had a long color-changing time (*ca.* 10 min) when exposed to hydrogen, and their manufacturing process is complex, making them unsuitable for large-scale industrial application.

Silicone rubber (SR) is widely used in gas separation membranes and gas permeability membranes because of its excellent processing performance, air permeability, chemical stability and permanent deformation resistance [26,27,28,29]. Prajapati [30] successfully prepared a novel ZIF-8/SR composite by introducing different amounts of ZIF-8 nanoparticles into the SR matrix. The highest C_3_H_8_ and C_3_H_6_ permeabilities, of 12,700 and 13,200 Barrer, respectively, were observed for these composites. Heidari [31] developed a carbon nanoparticle/SR composite that brought the maximum permeabilities of CO_2_ and C_3_H_8_ to 3584 and 11,026 Barrer, respectively, by increasing the affinity of gas to the membrane matrix. In addition, Shen [32] prepared a GO/SR composite that exhibits excellent C_3_H_8_ permeability, reaching the level of 1897 GPU. 

Our research team has conducted many investigations into hazardous gas detection, focusing particularly on hydrogen [33,34,35]. In our previous work, we developed a series of platinum-activated WO_3_ that demonstrated high hydrogen-sensitive gasochromic performance [36]. In this paper, utilizing this kind of WO_3_ as the hydrogen-sensitive nanoparticle, a set of WO_3_/SR composites were fabricated to achieve better application. The study systematically investigated the mechanical properties, hydrogen permeability, and gasochromic properties of the composites. The focus was on quantifying the gasochromic property of the composites to accurately evaluate the concentration of hydrogen. And a color scale was obtained based on the color of composites for easily assessing hydrogen concentrations in the environment. Furthermore, this paper presented a practical application case of the composite material at the hydrogen refueling stations, which provides an important reference for future research and application.

## 2. Results

### 2.1. Micromorphological Analyses

**Figure 1:**
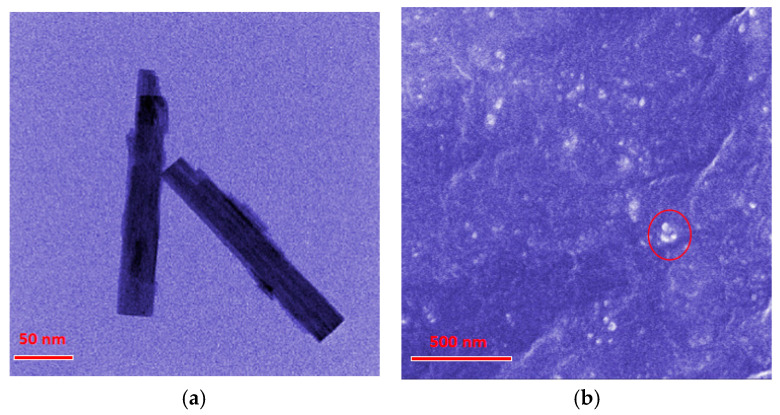
Electron microscope images (**a**) TEM of WO_3_ nanostructures. (**b**) SEM of the composites. White dots marked by red circle are WO_3_ nanostructures.

The micromorphology of the obtained WO_3_ nanostructures and of the composite materials was analyzed utilizing SEM and TEM methodologies. As shown in the Figure 1a TEM images, the WO_3_ nanostructures exhibited rod-like morphology with dimensions of 200 nm in length and 25 nm in diameter. The SEM image (Figure 1b) demonstrated the cross-sectional perspective of the composites, presenting an amorphous and compact continuous phase of SR matrix. Moreover, there were some white dots (marked with red circle) distributed in the SR continuous phase, which were proved to be WO_3_ nanostructures by EDS (Figure S1). This indicated that the WO_3_ nanostructures in the SR matrix were uniformly dispersed without significant aggregation.

### 2.2. Mechanical Properties

**Figure 2:**
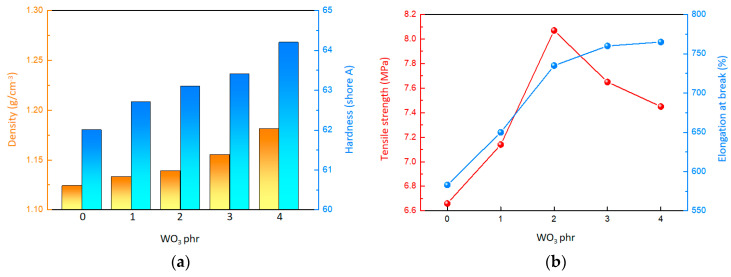
Mechanical properties of the composites. (**a**) Surface hardness and density. (**b**) Tensile property.

Based on Figure 2a and Table 1, it can be observed that both the density and surface hardness of the material increase as the WO_3_ content rises. Specifically, when the WO_3_ content is 0 phr, the material has a density of approximately 1.124 g/cm^3^ and a surface hardness of 62.0. However, when the WO_3_ content reaches 4 phr, the density of the material rises to around 1.181 g/cm^3^, and the surface hardness increases to 64.2. This trend indicates that the inclusion of WO_3_, serving as high-density inorganic fillers, contributes to the overall density and surface hardness of the material. According to Figure 2b and Figure S2, it is evident that the tensile strength of the material initially increases and then decreases with the gradual increment of WO_3_ content. When the WO_3_ content is 0 phr, the tensile strength is a mere 6.68 MPa. As the WO_3_ content is raised to 2 phr, the material reaches its peak tensile strength of 8.15 MPa. However, beyond this point, the tensile strength begins to decline, dropping to 7.46 MPa at the WO_3_ content of 4 phr. Concurrently, the elongation at break of the composites showed a steady increase. Despite the initial strengthening effect on tensile strength because of the use of WO_3_ as inorganic fillers, the crosslinking density of the composites could be impacted by the WO_3_, ultimately leading to a reduction in tensile strength.

**Figure 3:**
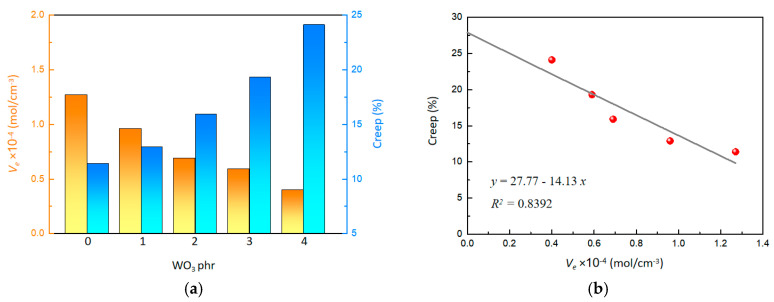
(**a**) Effect of WO_3_ content on *V_e_* and creep. (**b**) Effect of *V_e_* on creep.

The effect of WO_3_ on the crosslinking density (*V_e_*) is illustrated in Figure 3a. As the content of WO_3_ increased, the crosslinking density decreased from 1.27 to 0.40 mol/cm^3^, showing a clear downward trend. The decrease in crosslinking density can be attributed to the weakened crosslinking reaction of SR polymer chains, which is hindered by the mild acidic property of WO_3_. Furthermore, it is hard to establish strong physical entanglements between the WO_3_ nanostructures and the polymer chains of SR. As a result, the crosslinking density of the crosslinked network within the composite system is compromised.

Additionally, an improvement in creep was observed with increasing WO_3_ content (Figure 3a). As the WO_3_ content increased from 0 phr to 4 phr, the creep increased from 11.4% to 24.1%. A linear relationship between creep and crosslinking density was quantitatively determined (Figure 3b), as the following:

(1) y=27.77−14.13x

By extrapolating the crosslinking density to 0, an intercept value of 27.77 was obtained, indicating that the initial creep performance is affected by physical entanglement. This suggested that, as the WO_3_ content increases, the reduction in crosslinking density enhances creep performance. High creep means that polymer chains have better mobility, facilitating gas molecule penetration through the polymer matrix, which could help the material to exhibit excellent gasochromic characteristics.

### 2.3. Hydrogen Penetration Characteristics

**Figure 4:**
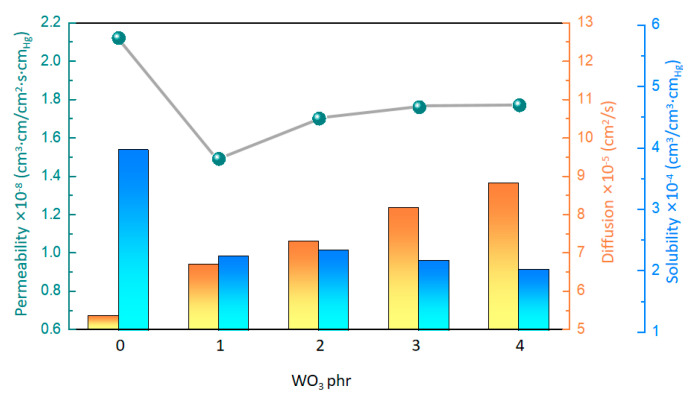
Hydrogen−permeability parameters of the composites with different WO_3_ contents.

The gas permeability of the material reflects the difficulty of small gas molecules passing through the material. For the gasochromic composite material, high gas permeability plays a crucial role in improving the gasochromic reaction between hydrogen and WO_3_. As shown in Table 2 and Figure 4 and Figure S3, the hydrogen permeability of the composites exhibited a nonlinear trend as the WO_3_ content gradually increased. At the WO_3_ content of 0 phr, the composite achieved a hydrogen penetration value of 2.12 cm^3^·cm/cm^2^·s·cm_Hg_. However, when the WO_3_ content reached 1 phr, a notable reduction in hydrogen permeability was observed, with a maximum value of 1.49 cm^3^·cm/cm^2^·s·cm_Hg_. Subsequently, the hydrogen permeability increased as the WO_3_ content further increased. At the WO_3_ content of 4 phr, the hydrogen permeability of the composite reached 1.77 cm^3^·cm/cm^2^·s·cm_Hg_.

**Figure 5:**
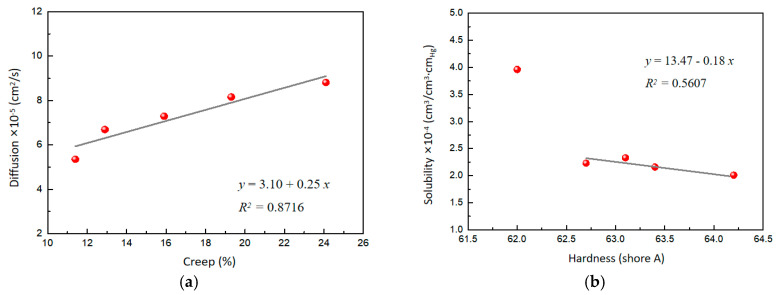
(**a**) Effect of creep on diffusion. (**b**) Effect of surface hardness on solubility.

Interestingly, the role of WO_3_ in the composites was very significant for hydrogen permeability. With the increasing content of WO_3_, the diffusion gradually increased, probably because WO_3_ changed the creep characteristics of the composites. Further analysis revealed a good linear correlation between the diffusion and creep, indicating that the increased mobility of the polymer chains contributes to the diffusion of hydrogen molecules in the polymer-based matrix (Figure 5a). On the other hand, the solubility of the composites gradually decreases with the increasing content of WO_3_. This phenomenon may be due to the increased surface hardness of the composites caused by WO_3_, resulting in the reduced solubility of hydrogen molecules on the surface. A correlation analysis of the solubility and the surface hardness also yields a linear equation (Figure 5b), which shows that the solubility of hydrogen gas molecules decreases with increasing surface hardness. Overall, WO_3_ significantly affects the hydrogen permeability of the composites by regulating the creep and surface hardness of the material.

### 2.4. Gasochromic Performance

**Figure 6:**
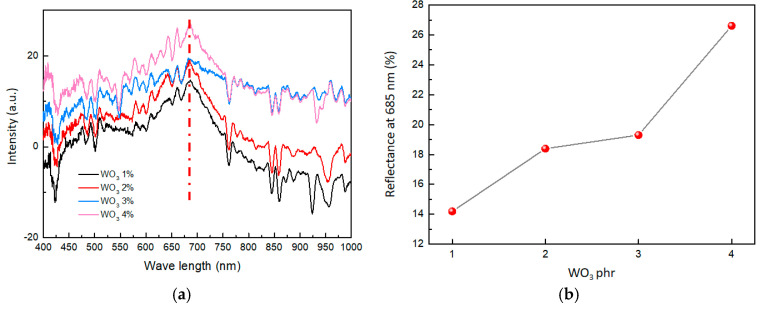
(**a**) Subtraction of visible light reflectance of composites with different WO_3_ contents. (**b**) Effect of WO_3_ contents on reflectance at 685 nm.

The color of a material’s surface is closely linked to its reflectance of visible light. In this paper, the variation in visible light reflectance is employed as a method to characterize changes in material color. By using a visible light monitor, the reflectance spectra of the composites were collected before and after hydrogen exposure. Then, the reflectance spectra of the composites before and after hydrogen exposure were subtracted to obtain the differential spectrum of visible light reflectance, depicting the coloring degree in the composite material (Figure 6a). According to Figure 6a, the largest differences in the subtraction of visible light reflectance were shown at 685 nm (the red dashed line). Therefore, the change in the color of the composites is primarily due to the change in the subtraction of visible light reflectance at 685 nm. 

As shown in Figure 6b, with a gradual increase in the content of WO_3_, the reflectance change of the composites also improved. When the WO_3_ content was 1 phr, the reflectance change was only 14.1%. However, when the WO_3_ content was increased to 4 phr, the reflectance change reached 26.7%. This suggested that as the content of WO_3_ increases in the composites, the reflectance change becomes stronger, affirming the more obvious color change of composites.

**Figure 7:**
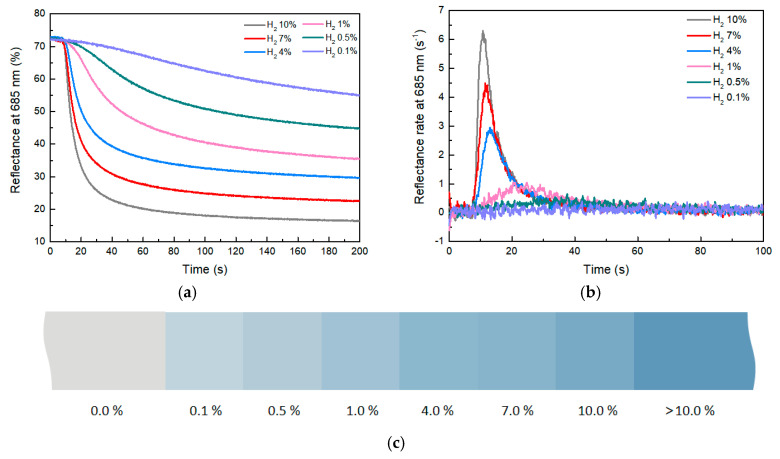
(**a**) Reflectance−change curve of composites with different hydrogen concentrations. (**b**) Reflectance−change−rate curve. (**c**) Color scale with different hydrogen concentrations.

The reflectance-change curve and the rate-of-change curve of composites with 3 phr WO_3_ at different hydrogen concentrations are shown in Figure 7a. As indicated by Figure 7b, the higher the hydrogen concentration, the greater the reflectance change and the faster the rate of reflectance change. When the hydrogen concentration is 10%, after approximately 40 s, the reflectance of the composite decreases from 72 to 23. This indicates a very noticeable color change of the material within 40 s, with a reflectance-change value of approximately 49 and a reflectance-change rate of 6.4% s^−1^. As the hydrogen concentration gradually decreases, the value and rate of reflectance change of the composite also decrease. When the hydrogen concentration dropped to 0.1%, the reflectance-change rate of the composite material decreased to 0.074% s^−1^, which represents the lower detection limit of the composite. These experimental results illustrate the impact of hydrogen concentration on the reflectance change of composites. High concentrations of hydrogen gas lead to rapid reflectance changes of composites, while low concentrations of hydrogen slow down the rate of reflectance change. This phenomenon could be related to chemical reactions between hydrogen and the composites.

With different concentrations of hydrogen, the reflectance of the composite will eventually remain stable without continuous changes, which can be used to indicate hydrogen concentration in the environment (Figure S4). This unique feature brings a way to look for leaks of colorless and odorless hydrogen gas via human vision. By employing the RGB color model to represent the reflectance of the composite at various hydrogen concentrations, a color gradient was constructed to visually represent different hydrogen concentrations (Figure 7c). This color scale made it possible to easily assess hydrogen concentrations in the environment based on the color of composites. This straightforward and practical approach can effectively find hydrogen leaks. 

**Figure 8:**
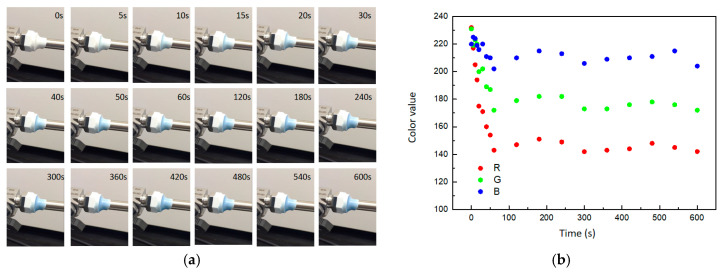
The gasochromic coloration of obtained composite. (**a**) Optical photos. (**b**) RGB model color change trend.

The composite was applied to cover the junction between a valve and a pipe, where a 1 mm leak was intentionally created, allowing for controlled hydrogen leakage at a rate of 1.0 mL/min. Color change of composites was monitored by video color analysis software. The results showed that with the hydrogen leaked, the composite gradually turned blue and eventually reached a steady color (Figure 8a). The RGB model color change trend was also shown in Figure 8b. During the initial phase of hydrogen leakage (<100 s), the values of the three channels in the RGB color model decreased rapidly. Channel A exhibited the most significantly change, dropping from 232 to 143, while channel B experienced the least variation, decreasing from 220 to 202. At the later stage of hydrogen leakage (>100 s), the values of the three color channels continued to fluctuate and tended towards stability. According to the comparison of composite color and the color scale (Figure 7c), the ambient hydrogen concentration had reached about 4% under the condition of 1.0 mL/min leakage, reaching the lower limit of a hydrogen explosion. The experimental results showed that the composite was instantly changing color as the hydrogen leaked and can quantitatively describe the ambient hydrogen concentration.

### 2.5. Practical Application

**Figure 9:**
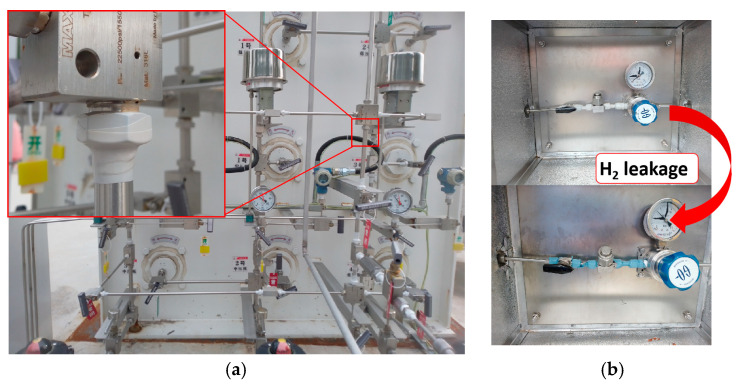
Photos of WO_3_/SR composite practical application. (**a**) Application details. (**b**) Color change of the composite before and after hydrogen leakage.

According to the above experimental results, the practical application of the optimized composite with 3 phr WO_3_ content was selected. The composites as disposable sensors have been tested in several Sinopec hydrogen refueling stations. In the hydrogen refueling station, all pipe joints and valves in the hydrogen compressor cabinets are wrapped in these composites, realizing the comprehensive coverage of the potential leakage parts (Figure 9a). During the process of hydrogen loading, once a hydrogen gas leak occurs, the composite will quickly change color from white to blue, accurately locating the leakage for the operator, which helps to eliminate the explosive danger in time (Figure 9b). Once the hydrogen leak has been repaired, the composite will gradually recover to its original color over a period of approximately 24 h in the air. The application of this composite provides a visual and quick hydrogen leakage detection method for operators, which guarantees the safety during the hydrogen loading process to support strongly the development of the hydrogen energy industry.

## 3. Materials and Methods

### 3.1. Materials

Hexagonal-phase WO_3_ nanostructures containing 1 wt% platinum were synthesized by the hydrothermal method, and the detailed information about WO_3_ was described in a previous paper [36]. The silicone rubber (SR) with a vinyl content of 0.1 mol% was provided by the Wynca Group, China. 2,4-Dichlorobenzoyl peroxide as the crosslinking agent was provided by Shanghai Macklin Biochemical Technology Co., Ltd., Shanghai, China. 

The formula of the composites is listed in Table 3. 

### 3.2. Preparation of the Composites

SR was carried out in an internal mixer for 0.5 min at 40 °C and 60 rpm. Following this, WO_3_ was added and mixed for 5 min. The obtained mixtures were then removed from the mixer and were added to the crosslinking agent on a laboratory two-roll mill with a roller speed ratio of 1:1.1. Subsequently, the mixtures were processed into sheets at 40 °C and then left at 25 °C for 24 h before curing. The prepared mixtures were subjected to a heated press at 140 °C for 7 min for curing, resulting in rubber sheets with a thickness of 1 mm.

### 3.3. Characterization and Measurements

**Figure 10:**
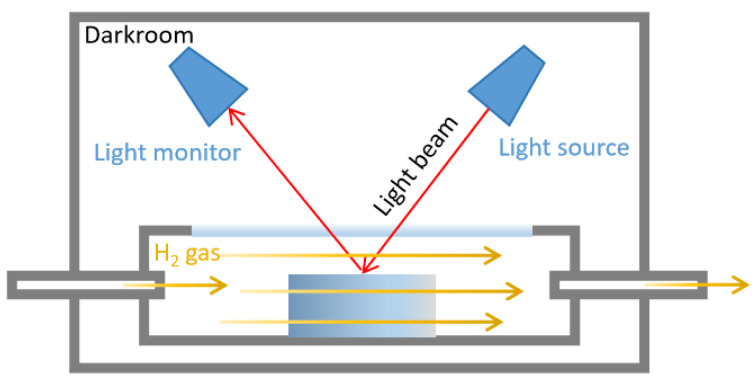
Schematic of in situ gasochromic sensing system for quantifying color change by visible light reflectance. (Red arrows represent the visible light path; Yellow arrows represent the hydrogen flow route).

Micromorphology images were observed by transmission electron microscopy (TEM, Talos F200i, Thermoscientific, Waltham, MA, USA) and a scanning electron microscope (SEM, Apreo 2S, Thermoscientific, USA). Tensile strength, elongation at break and creep were measured by an universal testing machine (i-Strentek1510, Labthink, Jinan, China) [37,38]. Crosslink density was measured by the solvent swelling method, using toluene as swelling solvent according to the reported methods [39]. The hydrogen permeability test was measured by a gas permeameter (VAC-V2, Labthink, China) [40]. Color changes were measured by an in situ gasochromic sensing system (HL-2000, Ocean Insight, Cardiff, UK), including a sample chamber, a darkroom, a light monitor, and a visible light source. The color change was quantified by the real-time difference in reflectance before and after H_2_ in dry synthetic air (Figure 10).

## 4. Conclusions

In this paper, the WO_3_/SR composite was prepared by a process of blending WO_3_ and silicone rubber. Through the use of TEM and SEM, it was observed that the WO_3_ rod-like nanostructures could be uniformly dispersed in the SR matrix. The influence of WO_3_ on the mechanical, hydrogen permeability and gasochromic properties of the composites was systematically analyzed. The hydrogen permeability of the composite reached 1.77 cm^3^·cm/cm^2^·s·cm_Hg_. When the hydrogen concentration was 10%, the visible light reflectance of the composite decreased from 72 to 23 in about 40 s, indicating a significant reflectance-change rate of 6.4% s^−1^. Furthermore, the composite could detect hydrogen concentrations as low as 0.1%, well below the explosion concentration. And a color scale was constructed by visible light reflectance change, which provided easy classification of hydrogen concentrations in the environment based on the color of composites. Finally, the composite materials as disposable sensors underwent testing at several Sinopec hydrogen refueling stations. The application of the composite offers a visual and quick method to detect hydrogen leakage for operators, effectively supporting the safety of hydrogen energy.

## Figures and Tables

**Table 1:** Mechanical properties of the composites with different WO_3_ content.

WO_3_ phr ^a^	Density (g/cm^3^)	Hardnesss (Shore A)	Tensile Strength (MPa)	Elongation at Break (%)	Crosslinking Density (mol/cm^3^)	Creep (%)
0	1.124	62.0	6.66	583	1.27	11.4
1	1.133	62.7	7.14	650	0.96	12.9
2	1.139	63.1	8.07	735	0.69	15.9
3	1.155	63.4	7.65	760	0.59	19.3
4	1.181	64.2	7.45	765	0.40	24.1

^a^ phr: parts per hundreds of rubber.

**Table 2:** Hydrogen permeability parameters of the composites.

WO_3_ phr ^a^	Permeability × 10^−8^ (cm^3^∙cm/cm^2^∙s∙cm_Hg_)	Diffusion × 10^−5^ (cm^2^/s)	Solubility × 10^−4^ (cm^3^/cm^3^∙cm_Hg_)
0	2.12	5.35	3.96
1	1.49	6.69	2.23
2	1.7	7.29	2.33
3	1.76	8.16	2.16
4	1.77	8.81	2.01

^a^ phr: parts per hundreds of rubber.

**Table 3:** Designations for the formulation of composites.

Ingredients/phr	1	2	3	4	5
SR	100	100	100	100	100
WO_3_	0	1	2	3	4

## Data Availability

The datasets generated during and/or analyzed during the current study are available from the corresponding author upon reasonable request.

## Supplementary Materials

The following supporting information can be downloaded at: https://www.mdpi.com/article/10.3390/molecules29153499/s1, Figure S1: EDS of the white dots in the SEM; Figure S2: Stress-strain curve of the composites with different WO_3_ contents; Figure S3: Hydrogen permeability curves of the composites with different WO_3_ contents; Figure S4: Trend of reflectance under low hydrogen concentration.
