# Supplementary material for: Characterization of WO3/Silicone Rubber Composites for Hydrogen-Sensitive Gasochromic Application
Source: Molecules. 2024 Jul 26;29(15):3499. doi: 10.3390/molecules29153499 (PMC11314044; doi:10.3390/molecules29153499)
Supplement: Supplementary file 1 [file molecules-29-03499-s001.zip › molecules-3109612-supplementary.pdf]

Supporting information

Characterization of WO_3/Silicone Rubber Composites for Hydrogen-Sensitive Gasochromic Application

Lin Wang [1,2], Ke Yang [2], Ping Yu [2], Huan Liu [2], Qingli Cheng [2], Anfeng Yu [2], Xinmei Liu [1,*] and Zhe Yang [2,*]

1 College of Chemistry and Chemical Engineering, China University of Petroleum (East China), Qingdao 266580, China; wangl.qday@sinopec.com
2 State Key Laboratory of Chemical Safety, Sinopec Research Institute of Safety and Engineering Co., Ltd., Qingdao 266000, China; kikiwow@163.com (K.Y.); yup.qday@sinopec.com (P.Y.); liuh.qday@sinopec.com (H.L.); chengql.qday@sinopec.com (Q.C.); yuaf.qday@sinopec.com (A.Y.)
* Correspondence: lxmei@upc.edu.cn (X.L.); yangzhe.qday@sinopec.com (Z.Y.)

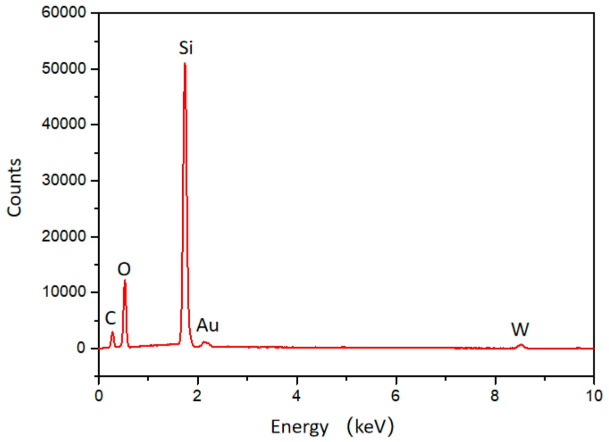

Figure S1 EDS of the white dots in the SEM

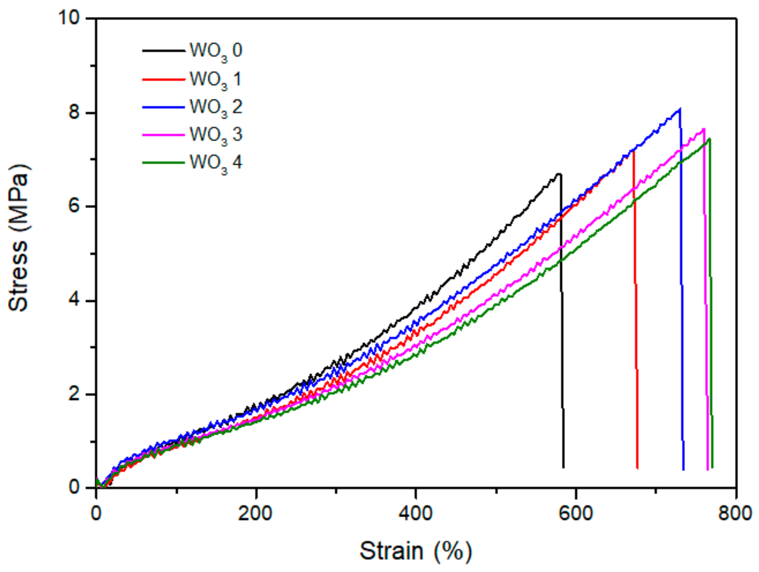

Figure S2 Stress-strain curve of the composites with different WO_3 contents

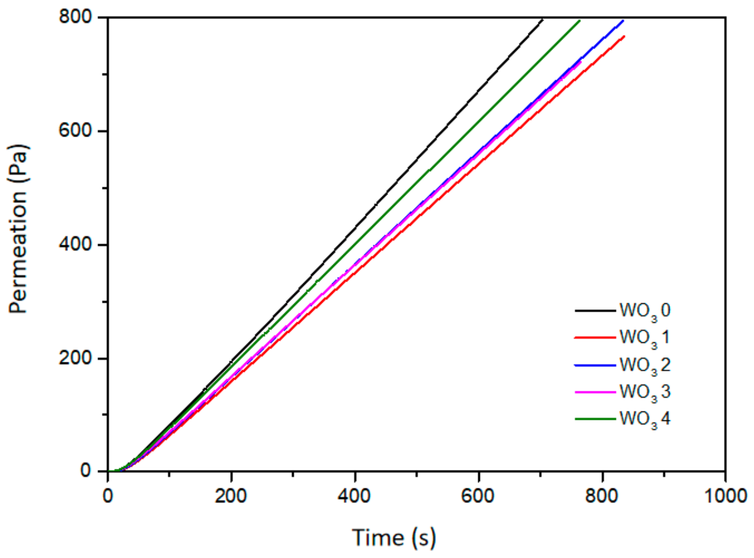

Figure S3 Hydrogen permeability curves of the composites with different WO_3 contents

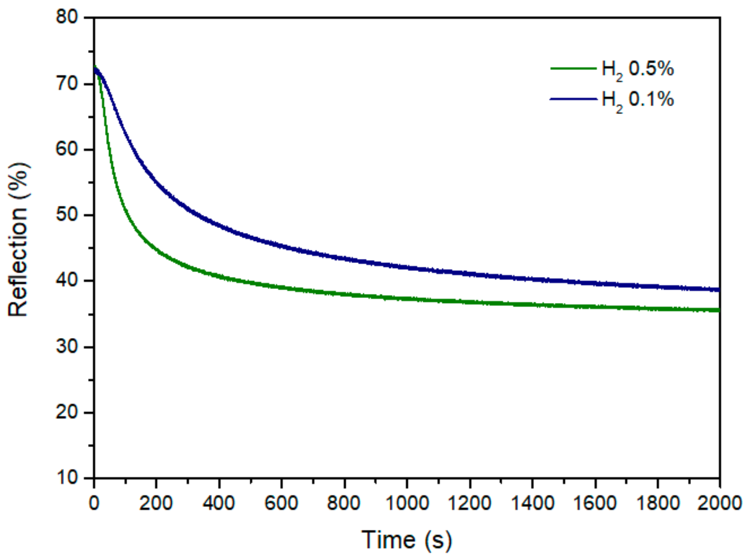

Figure S4 Trend of reflectance under low hydrogen concentration
